# A Rare Cause of Infantile-Onset Cardiomyopathy With Ocular Manifestations: Alström Syndrome

**DOI:** 10.7759/cureus.32857

**Published:** 2022-12-23

**Authors:** Rania Snobar, Madiha Mohamed, Ahmed AlKamali, Bhavna Gupta

**Affiliations:** 1 Pediatric Medicine, Al-Qassimi Women’s & Children’s Hospital, Sharjah, ARE; 2 Pediatric Medicine, Al-Qassimi Women's & Children's Hospital, Sharjah, ARE; 3 Pediatric Cardiology, Al-Qassimi Women's & Children's Hospital, Sharjah, ARE

**Keywords:** neonatal hypotonia, rare genetic disease, pendular nystagmus, restrictive cardiomyopathy, alström syndrome

## Abstract

We report a case of a five-month-old girl, who presented to our hospital with increased work of breathing, sweating since birth, and abnormal eye movements. On further evaluation, she was found to have restrictive cardiomyopathy, nystagmus, and hypotonia. Genetic workup showed a pathogenic variant in the ALSM1 gene, which confirmed the diagnosis of Alström syndrome. Alström syndrome is a rare condition that is characterized by a wide variety of multisystem manifestations, including visual disturbances, hearing impairment, and cardiomyopathy. This case report highlights Alström syndrome as one of the rare causes of early-onset infantile cardiomyopathy with nystagmus.

## Introduction

Alström syndrome is a rare autosomal recessive genetic disorder. It is caused by a mutation in the ALMS1 gene on chromosome 2. This is a multisystemic disorder characterized by cardiomyopathy, visual disturbance, respiratory disease, hepatic disease, insulin resistance, chronic renal disease, and hearing impairment [1]. One of the most common symptoms is cone-rod dystrophy leading to visual loss, with others being hearing loss and cardiomyopathy. While vision and hearing may be affected in these individuals, intelligence remains unaffected. Forty percent (40%) of children with Alström syndrome develop a dilated cardiomyopathy typically in infancy, and 20% develop restrictive cardiomyopathy, which eventually leads to congestive heart failure causing death [2].

## Case presentation

A five-month-old girl was referred to our facility with abnormal eye movements, left lower eyelid swelling since the age of three months, and increased work of breathing for one week. She had a background history of fast breathing and sweating during feeds since birth. She was born at term and had an uncomplicated perinatal history. However, her mother had a history of recurrent abortions. Her parents were first cousins, and there was no significant family history of neurological or cardiac diseases. In terms of her development, she had a mild motor delay but had a social smile and was interactive.

During the examination, she was conscious and alert with mild respiratory distress. Vertical nystagmus and left lower eyelid hemangioma were present. She had features of cardiac failure and short systolic murmur and hepatomegaly. There were generalized hypotonia and hyporeflexia.

**Figure 1:**
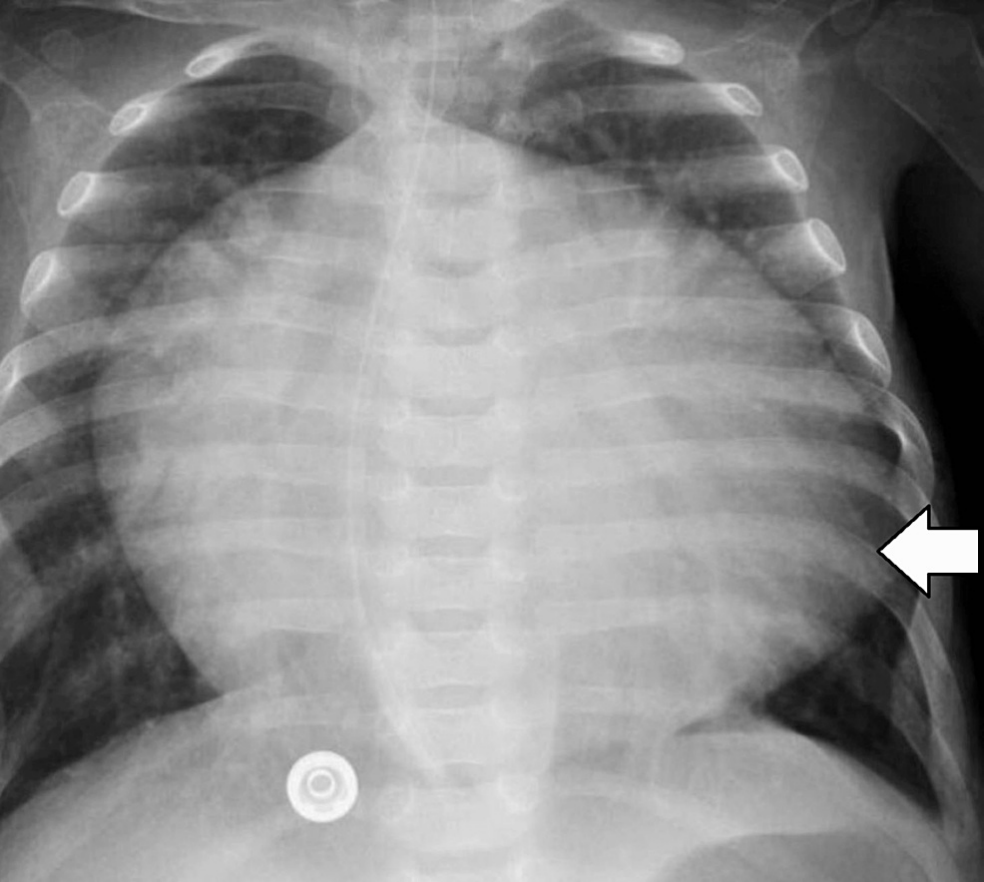
Chest X-Ray Revealing Cardiomegaly With a Narrow Vascular Pedicle and Congested Pulmonary Vasculature

A battery of investigations was carried out to find out the cause of her clinical condition. Basic lab investigations, including liver function and renal function tests, were normal. A chest X-ray revealed massive cardiomegaly and a narrow pedicle with congested pulmonary vasculature, as shown in Figure 1:

Echocardiography revealed restrictive cardiomyopathy with an ejection fraction of 65% and severe mitral and tricuspid regurgitation. MRI of the brain and magnetic resonance angiography (MRA) were both unremarkable but showed an intraconal, extraocular, soft tissue mass on the left side. The mass was large, defined, and lobulated, encasing the lateral rectus and inferior oblique muscles. This condition was suggestive of hemangioma. Fundus examination was unremarkable.

Metabolic conditions, lysosomal/ storage disorders, PHACE (posterior fossa anomalies, hemangioma, arterial anomalies, cardiac anomalies, and eye anomalies) syndrome, and related genetic conditions were the initial diagnostic considerations. The serum amino acid profile/very long chain fatty acids, urine test for organic acid disorders/mucopolysaccharidosis, and the serum acylcarnitine profile were normal. The skeletal survey was also unremarkable. Whole exome sequencing was then carried out, and it revealed a homozygous, likely pathogenic, variant in the ALSM1 gene. This finding was consistent with the diagnosis of Alström syndrome.

The patient had been under multidisciplinary team care and management in our hospital, but her clinical condition continued to deteriorate, and she succumbed to the disease.

## Discussion

Alström syndrome was initially described by a Swedish psychiatrist, Carl-Henry Alström, in 1959 [1]. This disorder is progressive in nature and has multi-systemic involvement. It is caused by autosomal recessive variants in the ALMS1 gene (Alström syndrome protein1). It is a rare disease with an approximate prevalence range from 1 in 10,000 to 1 in 1,000,000 patients [2]. After an extensive literature review, we have found around 1053 cases of this syndrome throughout the world so far [2].

In this disease, most tissues express low quantities of the ALMS1 protein, hence affecting multiple organ systems. The ALMS1 protein is necessary for ciliary structure and function, ciliary signaling pathways, intracellular trafficking, cell differentiation, and metabolic homeostasis, albeit its biological activity is unclear [2]. Hence, it is considered a ciliopathy and classified with other ciliopathies like Bardet-Biedl and Joubert syndromes. Around 268 pathogenic variants have been involved in ALMS, and 96% of them are insertions and deletions [2].

The phenotypical expression of the disease, its severity, and the age of presentation of its various features can vary significantly among patients, and this variation is found even within families. This syndrome is characterized by cardiomyopathy, visual and hearing impairment, extreme insulin resistance and related complications, and renal dysfunction. The initial presentation occurs as early as four months of life, usually as nystagmus or infantile-onset cardiomyopathy. It is estimated that about 20% of children develop restrictive cardiomyopathy, usually between early adolescence and adulthood while 40% of patients have dilated cardiomyopathy, usually in infancy. Congestive cardiac failure, a complication of cardiomyopathy, affects more than 60% of individuals [2]. Both congestive cardiac failure and cardiomyopathy can lead to death in these patients. Although cardiomyopathy may not be present initially, diagnosis can assist in managing this deadliest feature early on [2,3].

Another significant manifestation, in general, for all patients with an ALMS1 gene mutation below two years, is cone-rod dystrophy, which commonly occurs between birth and 15 months of age. It has been reported that bilateral sensorineural hearing loss appears in a significant proportion of cases in infancy [3]. Hearing and vision impairment can lead to developmental delays in these children. Although there is a developmental delay, cognitive impairment is relatively uncommon. Childhood obesity occurs in most patients, and almost all individuals are prone to insulin resistance. This syndrome may also affect the respiratory system, with features like bronchial infections, pulmonary hypertension, and fibrosis.

There may also be hepatic involvement in this syndrome, causing portal hypertension, fibrosis, cirrhosis, and liver failure. These features usually manifest themselves after childhood. Renal involvement can occur between adolescence and adulthood, and it can cause glomerulosclerosis, which can ultimately lead to renal failure. Alström syndrome is relentlessly progressive and can lead to premature death [2].

Although it is a retinal disease typically causing infantile nystagmus, a brain MRI is the most frequent initial test for children with nystagmus [2]. A complete eye examination, electroretinogram, optical coherence tomography, and molecular genetic testing are ideal for children who present with nystagmus only. Nevertheless, these tests have their limitations due to lack of access, availability, risks, and cost of sedation. In our patient, these investigations could not be done, hence ophthalmology examination did not reveal anything significant.

The only way to confirm the diagnosis of Alström syndrome is a molecular genetic study because there is no biochemical, histological, or imaging test to do so [3]. Due to the premature mortality of most affected children, the diagnosis is likely to be underestimated in the absence of routine genetic tests. Until now, there is no treatment available for this syndrome. Hence, a multidisciplinary team with various specialties in a specialist center, working closely with community care providers, is necessary for optimal disease management [3,4].

## Conclusions

Alström syndrome is a rare genetic disorder caused by mutations in the ALMS1 gene. It affects multiple organ systems in the body, and because of its progressive nature, can lead to significant morbidity and mortality in patients. The lack of available knowledge about this rare and complex syndrome may lead to misdiagnosis or delayed diagnosis and suboptimal care of patients. We have reported this case to make clinicians aware of Alström syndrome, as this can be one of the causes of early onset-infantile cardiomyopathy with nystagmus. Although no treatment is available for this syndrome, early recognition can help initiate multidisciplinary care for these children and improve their quality of life.

## References

[1] (2016). Alström syndrome. NORD. https://rarediseases.org/rare-diseases/alstrom-syndrome/.

[2] Etheridge T, Kellom ER, Sullivan R, Ver Hoeve JN, Schmitt MA (2020). Ocular evaluation and genetic test for an early Alström syndrome diagnosis. Am J Ophthalmol Case Rep.

[3] Tahani N, Maffei P, Dollfus H (2020). Consensus clinical management guidelines for Alström syndrome. Orphanet J Rare Dis.

[4] Alström Syndrome UK. What is Alström syndrome?. http://www.alstrom.org.uk/what-is/.

